# Airborne Aerosol Generation During Endonasal Procedures in the Era of COVID-19: Risks and Recommendations

**DOI:** 10.1177/0194599820931805

**Published:** 2020-05-26

**Authors:** Alan D. Workman, Aria Jafari, D. Bradley Welling, Mark A. Varvares, Stacey T. Gray, Eric H. Holbrook, George A. Scangas, Roy Xiao, Bob S. Carter, William T. Curry, Benjamin S. Bleier

**Affiliations:** 1 Department of Otolaryngology, Massachusetts Eye and Ear Infirmary, Boston, Massachusetts, USA; 2 Harvard Medical School, Boston, Massachusetts, USA; 3 Department of Neurosurgery, Massachusetts General Hospital, Boston, Massachusetts, USA

**Keywords:** COVID-19, airborne, aerosolization, endoscopy, nasal endoscopy, aerosol-generating surgery, aerosol-generating procedure

## Abstract

**Objective:**

In the era of SARS-CoV-2, the risk of infectious airborne aerosol generation during otolaryngologic procedures has been an area of increasing concern. The objective of this investigation was to quantify airborne aerosol production under clinical and surgical conditions and examine efficacy of mask mitigation strategies.

**Study Design:**

Prospective quantification of airborne aerosol generation during surgical and clinical simulation.

**Setting:**

Cadaver laboratory and clinical examination room.

**Subjects and Methods:**

Airborne aerosol quantification with an optical particle sizer was performed in real time during cadaveric simulated endoscopic surgical conditions, including hand instrumentation, microdebrider use, high-speed drilling, and cautery. Aerosol sampling was additionally performed in simulated clinical and diagnostic settings. All clinical and surgical procedures were evaluated for propensity for significant airborne aerosol generation.

**Results:**

Hand instrumentation and microdebridement did not produce detectable airborne aerosols in the range of 1 to 10 μm. Suction drilling at 12,000 rpm, high-speed drilling (4-mm diamond or cutting burs) at 70,000 rpm, and transnasal cautery generated significant airborne aerosols (*P* < .001). In clinical simulations, nasal endoscopy (*P* < .05), speech (*P* < .01), and sneezing (*P* < .01) generated 1- to 10-μm airborne aerosols. Significant aerosol escape was seen even with utilization of a standard surgical mask (*P* < .05). Intact and VENT-modified (valved endoscopy of the nose and throat) N95 respirator use prevented significant airborne aerosol spread.

**Conclusion:**

Transnasal drill and cautery use is associated with significant airborne particulate matter production in the range of 1 to 10 μm under surgical conditions. During simulated clinical activity, airborne aerosol generation was seen during nasal endoscopy, speech, and sneezing. Intact or VENT-modified N95 respirators mitigated airborne aerosol transmission, while standard surgical masks did not.

The COVID-19 pandemic has catalyzed an unparalleled disruption in the provision of health care around the world. Following its detection in December 2019, health policy shifted from an initial strategy of containment to mitigation.^1^ These efforts have been largely successful at preventing hospital resources from becoming overwhelmed within the United States. However, it has required the delay or cancellation of almost all elective patient visits and procedures. Fortunately, infection and case fatality rates have begun to plateau in even the most severely affected regions. Clinicians and hospitals now face challenging decisions regarding how to safely allow elective patients back into the clinics and operating rooms. This difficulty in planning is compounded by a persistent lack of personal protective equipment, effective treatments for COVID-19, COVID-19 testing capacity and turnaround time, and clarity regarding sensitivity and specificity of the currently available tests for COVID-19.^2^

Rhinologic cases are of unique concern in this reopening phase. Delays in elective care do appear to be associated with worse outcomes^3^ and higher costs.^4^ However, endoscopic procedures have been shown to carry a risk of respiratory droplet formation in diagnostic and surgical settings.^5^ While these risks can be mitigated with low-level personal protection equipment, the potential of airborne aerosol generation during endoscopic procedures has not been studied. An evidence-based analysis of this potential is essential because it bears directly on the status of endonasal instrumentation as an aerosol-generating procedure (AGP) with its attendant heightened requirements for personal protective equipment, air handling, and environmental controls.

The purpose of this study was to therefore (1) quantify airborne aerosol production following endonasal instrumentation during cadaveric surgical and clinical diagnostic conditions and (2) determine the relative efficacy of source control solutions.

## Methods

### Study Design

The surgical simulation was Institutional Review Board approved through a formal excess tissue protocol. The clinical simulation was reviewed by the Partner’s Human Research Committee director and performed under the Quality Improvement Initiative at Massachusetts Eye and Ear; as such, it was not required to be formally supervised by the Institutional Review Board per its policies. All cadaver experiments in this study were performed in a dedicated surgical laboratory with 2 fresh-frozen head specimens at room temperature. The clinical examination room (111 sq ft) and the surgical laboratory (726 sq ft) were equipped with air exchangers operating at a rate of 6 total air changes per hour.

### Aerosol Sampling

Aerosol sampling was performed with an optical particle sizer (OPS 3330; TSI Inc), which measures particle number, concentration, and size distribution with single particle–counting technology up to a size of 10 μm. Flow rate through the OPS 3330 is a constant 1.0 L/min via a 3-mm port. Particle size distribution is measured in 16 user-adjustable channels. Total particle counts by size over a period of timed data were collected.

### Surgical Simulation

**Figure 1.**
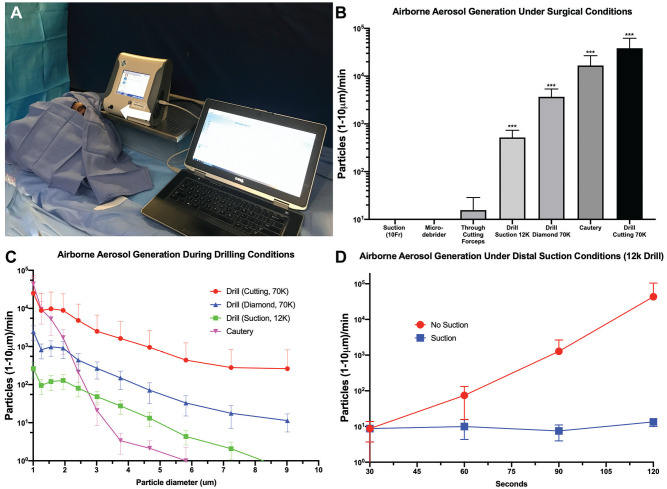
Surgical simulation: (A) Experimental setup (arrow denotes intake port). (B) Aerosol generation after 2 to 5 minutes. ***P < .001. (C) Particles separated by size (1-10 µm). (D) Aerosols in the presence and absence of distal tip suction. Values are presented as mean ± SE.

The cadaver head was placed in a supine position with the nostril situated 15 cm from the optical particle sizer (OPS) intake port (**Figure 1A**). Five milliliters of saline was irrigated into the nose with a syringe prior to each surgical condition. For surgical visualization, a high-definition endoscopic camera was attached to a 4-mm 0° endoscope (Karl Storz). Background samplings were obtained prior to surgical conditions, and at least 2 minutes elapsed between experiments to allow for verification of return to baseline aerosol concentrations at the intake port. Suction was utilized to evacuate any retained intranasal particulates following all drilling and cautery conditions. Experiments were conducted in 30-second durations with sequential replicates performed for a total of 2 to 5 minutes. The surgical conditions included (1) nasal suctioning with a 10Fr Frazier suction; (2) hand-actuated instrumentation with through-cutting forceps of the middle turbinate; (3) powered suction microdebridement (4-mm Tricut blade at 5000 oscillations/min; Medtronic) of the posterior nasal septum; (4) powered high-speed drilling of the sphenoid rostrum with a 4-mm diamond reverse taper suction drill at 12,000 rpm (Medtronic); (5) powered high-speed drilling of the sphenoid rostrum with a Midas Rex Legend Stylus with 4-mm diamond bur at 70,000 rpm (Medtronic); (6) powered high-speed drilling of the sphenoid rostrum with a Midas Rex Legend Stylus with 4-mm cutting bur at 70,000 rpm; and (7) battery-powered endonasal cautery of the inferior turbinate (Acu-Tip; Practicon). Each intervention was performed in duplicate on 2 separate cadaver heads.

### Clinical Simulation

**Figure 2.**
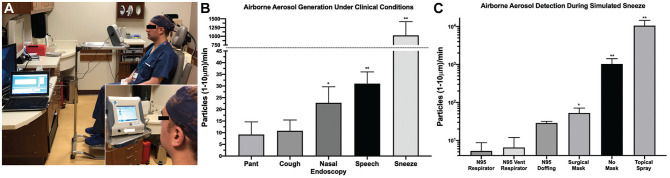
Clinical simulation: (A) Experimental setup (arrow denotes intake port). (B) Airborne aerosol generation during simulated clinical conditions. (C) Airborne particle generation under sneeze conditions with various source controls. *P < .05. **P < .01. Values are presented as mean ± SE.

Participants were seated upright in a clinical room examination chair with the naris placed 15 cm from the OPS intake port (**Figure 2A**). Background samplings were obtained in an empty clinic room, and at least 2 minutes elapsed between experiments to allow for return to baseline aerosol concentrations at the intake port. Each experiment was conducted in 30-second durations with sequential replicates performed for a total of 1 minute. The clinical conditions included (1) simulated heavy mouth breathing (eg, panting) with breaths every 3 seconds; (2) simulated coughing every 5 seconds; (3) speech by reading of the “Rainbow Passage,” a standardized vocalization paradigm (Voice and Articulation Drillbook; Harper & Row); (4) simulated sneezing every 10 seconds; (5) simulated nasal endoscopy by the intranasal placement of a 2.7-mm 0° rigid and 3.5-mm flexible endoscope (Karl Storz) for 20 seconds, followed by removal; and (6) simulated topical spray of a 1% lidocaine and oxymetazoline 0.05% solution (1:1, MADomizer; Teleflex) 15 cm away from the OPS intake port every 10 seconds. Participants took a sip of water in between each condition to ensure adequate and consistent hydration. Each intervention was performed in duplicate on 2 participants.

Following behavioral simulation, participants then performed additional simulated sneezing every 10 seconds for 30-second replicates with the opening of the mouth positioned 15 cm from the OPS intake port while wearing (1) a standard level 1 surgical mask (Halyard Health), (2) N95 Health Care Particulate Respirator and Surgical Mask (1860; 3M), and (3) modified N95 VENT respirator (valved endoscopy of the nose and throat) as previously described,^5^ to allow passage of an endoscope through the mask while maintaining a tight seal. An additional trial was performed by doffing of the N95 respirator for 30 seconds following sneezing to measure airborne aerosol release after mask removal.

### Statistical Analysis

Stata 13 (StataCorp) was used for statistical analysis to assess differences between background particle concentration and particles generated during simulated clinical and surgical activities. Nonparametric statistical techniques were utilized due to small sample sizes, with Bonferroni correction for multiple comparisons. Average background particle concentration (separate for clinical encounter and surgical laboratory encounter) was subtracted from each condition prior to data visualization as previously described.^6^ Prism Version 8 (GraphPad Software) was used for visualization of data.

## Results

### Surgical Simulation

#### Airborne Aerosol Generation During Cold Instrumentation and Microdebridement

All sampling periods were 30 seconds, and conditions were performed in duplicate with 2 cadaver heads. Sixteen background samples were obtained, as spaced between experiments, and minimal variability in background was observed. (1) Nasal suctioning with a 10Fr Frazier suction for 4 sampling periods and (2) endoscopic through biting of the middle turbinate (hand actuated) for 10 sampling periods did not produce significant detectable airborne aerosols in the range of 1 to 10 μm (**Figure 1B**). Application of a microdebrider to the posterior septum with debridement of tissue and declogging external to the nare did not produce 1- to 10-μm airborne aerosols over 10 sampling periods (5 minutes). The cutting edge of the microdebrider was open upon introduction and removal.

#### Airborne Aerosol Generation During High-Speed Drilling Conditions

With the cadaver head in the surgical position, 3 separate drilling conditions were performed: (1) a suction drill at 12,000 rpm for ten 30-second samples, (2) a powered high-speed drill at 70,000 rpm with a 4-mm diamond bur for four 30-second samples, and (3) a powered high-speed drill at 70,000 rpm with a 4-mm cutting bur for four 30-second samples. The drill was used to remove bone at the sphenoid rostrum. In all 3 conditions, significant airborne aerosol generation in the range of 1 to 10 μm was observed (**Figure 1B**; suction drill, *P* < .001, *U* = 15, n = 20; diamond drill, *P* < .001, *U* = 0, n = 8; cutting drill, *P* < .001, *U* = 1.5, n = 8; Mann-Whitney *U* test). Particle generation was observed to increase throughout the duration of the drilling, with increased particle generation during the latter portion of drilling periods. Particle number decreased with increasing particle diameter across the 1- to 10-μm range (**Figure 1C**). Finally, an additional experiment was performed demonstrating increased particle generation in the absence of suction with the suction drill at 12,000 rpm over the first 120 seconds of drilling (**Figure 1D**).

#### Airborne Aerosol Generation During Transnasal Cautery

Transnasal cautery of the inferior turbinate demonstrated significant particle generation in the range of 1 to 10 μm over background in four 30-second samples (**Figure 1B**; *P* < .001, *U* = 0, n = 8; Mann-Whitney *U* test). Particles generated were on average smaller than those observed in the drilling conditions (**Figure 1C**).

### Clinical Simulation

#### Airborne Aerosol Generation During Simulated Patient Activities

Participants were positioned sitting upright with the nose and mouth 15 cm from the aperture of the OPS air intake valve. All samples were collected over a period of 30 seconds and performed with 2 participants and at least 2 replicates per participant (n = 4-10). Panting and coughing generated detectable 1- to 10-μm aerosols that were not significantly greater than background (**Figure 2B**). Nasal endoscopy and speech conditions generated significant airborne aerosols (nasal endoscopy, *P* < .05, *U* = 10, n = 8; speech, *P* < .01, *U* = 6.5, n = 10; Mann-Whitney *U* test). Simulated sneezing generated the most airborne particles per minute by an order of magnitude (*P* < .01, *U* = 0, n = 4; Mann-Whitney *U* test). Simulated topical spraying of lidocaine and oxymetazoline generated airborne aerosols comparable to those generated with sneezing (**Figure 2C**; *P* < .01, *U* = 0, n = 4; Mann-Whitney *U* test).

#### Airborne Aerosol Detection During Simulated Sneeze Under Masked Conditions

As simulated sneezing generated the largest number of 1- to 10-μm airborne aerosols, several sneezing conditions were performed with different source control mask solutions. The surgical mask alone attenuated airborne aerosol generation (**Figure 2C**); however, statistically significant aerosol escape was still detected (*P* < .05, *U* = 2, n = 4; Mann-Whitney *U* test). An N95 respirator and a modified N95 VENT respirator ameliorated airborne particle generation to background levels. N95 doffing following simulated sneezing over a 30-second period demonstrated an increase in airborne particle generation that did not reach significance above background.

## Discussion

While droplet and contact infectious transmission in SARS-CoV-2 has been largely accepted, the role of airborne transmission remains unclear. This mode is of particular concern in the health care setting given the propensity for AGPs to produce particles <10 µm.^7^ The size of the SARS-CoV-2 virus is approximately 60 to 140 nm, based on electron micrographs.^8^ Since the advent of COVID-19, the field of otolaryngology has found itself grappling with potential aerosolization risk of endoscopic procedures despite a distinct lack of quantitative evidence to guide best practices. In an effort to address this unmet need, our team previously reported on a semiquantitative method to determine the risk of droplet aerosol production during outpatient diagnostic and surgical endonasal procedures.^5^ The purpose of the current study was to extend those findings into the range of airborne aerosols.

Our surgical simulation conditions were designed to test a variety of endonasal instruments from suction and through-cutting forceps to powered devices and thermal cautery. Our findings were generally consistent with our prior study in that use of a surgical drill carried the greatest risk of generating detectable aerosols. The concomitant use of suction appeared to provide some benefit in reducing aerosol concentration; however, the lower speed of the suction drill is a confounding variable. Similarly, the microdebrider with distal tip suction did not produce detectable aerosols, even when requiring removal and active unclogging adjacent to the detector. Conversely, thermal cautery produced significant and particularly fine aerosols, which is consistent with the previous literature.^9^ These findings serve to provide further evidence that drills and cautery remain the endonasal surgical procedures of greatest risk.

With regard to the clinical diagnostic conditions, our findings demonstrated that detectable airborne aerosols are generated during limited periods of speech, panting, cough, and sneeze. However, talking and sneezing were the only behaviors associated with a significant increase over background. Unfortunately, the most common method used to reduce sneezing—namely, topical nasal anesthesia and decongestion spray—also produced a significant number of aerosols. While the lack of significance in the other behavioral conditions could be attributed to the short testing duration and use of healthy volunteers, these results are consistent with prior physiologic reports confirming the differential risk of speech and sneeze conditions.^10-13^ Unlike our prior droplet data,^5^ nasal endoscopy was associated with airborne aerosol production irrespective of whether a rigid or flexible scope was utilized. AGPs are defined by the Centers for Disease Control and Infection as “commonly performed medical procedures . . . that create uncontrolled respiratory secretions.” Insofar as endoscopic examinations (1) require prolonged close proximity to the patient, (2) produce detectable airborne aerosols, and (3) carry a distinct yet unpredictable risk of triggering sneeze events, our findings suggest that nasal endoscopy carries a similar risk profile as currently recognized AGPs.^7,14^

Our tested mask conditions focused on the ability to mitigate sneeze-associated aerosol production, as this was clearly the behavior of greatest risk. The existing literature regarding the utility of masks is complex, as studies tend to focus on discrete attributes, such as filtration efficiency, performance under steady and episodic conditions, and the relationship between mask use and infectious transmission. Epidemiologic and virologic studies have suggested that surgical masks may be equivalent to N95 respirators at protecting health care workers from infectious respiratory viruses.^15,16^ Similarly, some virologic reports have shown that surgical masks alone are adequate to prevent coronavirus aerosol spread in the droplet and airborne ranges during talk and cough conditions.^17^ Conversely, studies employing episodic stresses such as sneeze have shown that surgical masks are vulnerable to leakage from dynamic changes in pressure and air velocity.^16,18,19^ This is perhaps not surprising as sneezing may produce thousands of airborne droplet nuclei at high speeds.^12,13^ The evident discrepancies among mask efficacy readouts highlights the importance of context-dependent testing as a basis for the creation of subspecialty-specific safety guidelines. Our results were consistent with previous findings^16,18,19^ in that an intact surgical mask was incapable of controlling the spread of sneeze-associated airborne aerosols. This result stands in contrast to our prior findings in which a surgical mask did prevent simulated respiratory droplet contamination.^5^ Conversely, the N95 respirator in the intact and VENT modification conditions appeared to effectively contain aerosol spread. Though not statistically significant, some contamination occurred after N95 respirator removal, suggesting that when used as source control, masks should not be doffed within the clinical space.

As we apply these data to infection prevention and control recommendations in the outpatient otolaryngology setting, it is useful to conceptualize the protection needs of the 3 *P*’s—namely, the patient, the provider team (including administrative and medical staff), and the physical plant (including the clinic/waiting room surfaces and air supply). Comprehensive adherence to “standard precautions” as defined by the Centers for Disease Control and Prevention^14^ will tend to simultaneously address each of these groups and should integrate source, engineering, and environmental control strategies. Our results suggest that the proper use of a fit-tested N95 or equivalent VENT respirator is effective at mitigating sneezing, the behavior associated with the highest number of aerosols at the highest velocities. Consequently, these latter barrier strategies may be considered (1) a source control by protecting the provider/physical plant from the patient and (2) an engineering control by protecting the patients from the providers and one another.

There are several limitations to this study that bear discussion. As the surgical simulation was performed in a cadaver head, it is possible that the lack of pulsatile blood supply at body temperature and physiologic mucus secretion may alter the propensity for aerosol production in the range of 1 to 10 μm. Consequently, further studies during active surgery are warranted. It is important to note that this study specifically measured optical particle size and did not use an aerodynamic particle sizer or alternative instrument to measure the aerodynamic nature of these particles, material makeup, particle volume, shape, density, or rate of settling. Particulate observed in any condition is known to be present only at the distance measured from the source of generation and was measured in real time; this does not reflect particulate desiccation or morphological changes that may occur over time, or settling rates of these particles. Future studies should investigate the aerodynamic properties of these particles to determine their likelihood of being deposited within the upper respiratory tract. With regard to the testing of the clinical diagnostic conditions, we must stress that our methodology was sensitive only to the generation of airborne droplet nuclei. The study was not designed to detect the presence of virus within these particles or their infectious transmissibility. However, in the absence of clear data on the minimum infectious dose of SARS-CoV-2, we believe that our findings should be interpreted in the most conservative context possible with respect to infectious control recommendations.

## Conclusion

Our study represents a systematic effort to quantify the degree of airborne aerosol production associated with a variety of endonasal procedures. The surgical simulation data confirm that the use of high-speed drills and cautery produces the largest number of particles. The clinical conditions revealed that endoscopic instrumentation, speech, and sneezing all produced significant detectable airborne aerosols within only 30 seconds of measurement. An intact surgical mask failed to fully protect against sneeze-associated contamination. Therefore, surgical VENT masks, as previously described by our group, may not be sufficient in terms of <10-µm particles. However, when applied to an N95 respirator, the VENT modification retained the ability contain airborne aerosols. These results suggest that while nasal endoscopy carries a risk profile similar to established AGPs, barrier mask solutions offer the potential of effective source and engineering controls.
